# CRISPR interference (CRISPRi) for gene regulation and succinate production in cyanobacterium *S. elongatus* PCC 7942

**DOI:** 10.1186/s12934-016-0595-3

**Published:** 2016-11-15

**Authors:** Chun-Hung Huang, Claire R. Shen, Hung Li, Li-Yu Sung, Meng-Ying Wu, Yu-Chen Hu

**Affiliations:** Department of Chemical Engineering, National Tsing Hua University, Hsinchu, 30013 Taiwan

**Keywords:** CRISPRi, PCC 7942, Cyanobacteria, sgRNA, Metabolic engineering, Gene regulation

## Abstract

**Background:**

Cyanobacterium *Synechococcus elongatus* PCC 7942 holds promise for biochemical conversion, but gene deletion in PCC 7942 is time-consuming and may be lethal to cells. CRISPR interference (CRISPRi) is an emerging technology that exploits the catalytically inactive Cas9 (dCas9) and single guide RNA (sgRNA) to repress sequence-specific genes without the need of gene knockout, and is repurposed to rewire metabolic networks in various procaryotic cells.

**Results:**

To employ CRISPRi for the manipulation of gene network in PCC 7942, we integrated the cassettes expressing enhanced yellow fluorescent protein (EYFP), dCas9 and sgRNA targeting different regions on *eyfp* into the PCC 7942 chromosome. Co-expression of dCas9 and sgRNA conferred effective and stable suppression of EYFP production at efficiencies exceeding 99%, without impairing cell growth. We next integrated the dCas9 and sgRNA targeting endogenous genes essential for glycogen accumulation (*glgc*) and succinate conversion to fumarate (*sdhA* and *sdh*B). Transcription levels of *glgc*, *sdhA* and *sdh*B were effectively suppressed with efficiencies depending on the sgRNA binding site. Targeted suppression of *glgc* reduced the expression to 6.2%, attenuated the glycogen accumulation to 4.8% and significantly enhanced the succinate titer. Targeting *sdhA* or *sdhB* also effectively downregulated the gene expression and enhanced the succinate titer ≈12.5-fold to ≈0.58–0.63 mg/L.

**Conclusions:**

These data demonstrated that CRISPRi-mediated gene suppression allowed for re-directing the cellular carbon flow, thus paving a new avenue to rationally fine-tune the metabolic pathways in PCC 7942 for the production of biotechnological products.

**Electronic supplementary material:**

The online version of this article (doi:10.1186/s12934-016-0595-3) contains supplementary material, which is available to authorized users.

## Background

Cyanobacteria are photoautotrophic procaryotes that can exploit sunlight and CO_2_ as the sole energy and carbon sources to convert CO_2_ into organic compounds via photosynthesis [1]. Many cyanobacterial strains are amenable to natural transformation and homologous recombination for gene manipulation. Thanks to these attributes, genetically engineered cyanobacteria have drawn increasing attention as a chassis for the production of biofuels and bio-derived chemicals [2]. For instance, *Synechococcus elongatus* PCC 7942 has been genetically modified as a “cell factory” to divert native metabolic pathways to produce 2,3-butanediol, 2-methyl-1-butanol, isopropanol, free fatty acid, 1,2-propanediol, isopropanol, isobutyraldehyde and isobutanol, etc. [3].

Typically, PCC 7942 is engineered by knocking in genes encoding synthetic pathways and/or knocking out genes in competing pathways [4, 5]. However, generation of a single gene knockout mutant may take >3 weeks using conventional methods [6] due to its long doubling time and oligoploidy nature [1]. Sometimes deletion of certain genes essential for metabolic balances is not feasible or easily achieved as the deletion might be lethal to the cells. Furthermore, in many cases intermediate levels of enzyme expression may result in better product titer [7]. Therefore, tunable and balanced gene expression is desirable for high productivity, product titer, and conversion yield, and controllable gene repression/knockdown may be preferable than gene deletion for certain biotechnological applications and synthetic biological manipulations [8].

CRISPR-Cas9 is a newly developed RNA-guided genome editing system [9, 10]. CRISPR-Cas9 system comprises the Cas9 nuclease, transacting RNA (tracrRNA) and CRISPR RNA (crRNA). crRNA/tracrRNA complexes with Cas9 and, guided by the spacer sequence on crRNA, orchestrate to recognize protospacer-adjacent motif (PAM) and bind to proximal complementary sequence. After the recognition and binding, Cas9 nuclease triggers double strand break (DSB) at the chromosomal DNA [11]. Coupled with an editing template DNA, such CRISPR-Cas9-mediated DSB is exploited for programmable genome engineering of diverse cell types and model organisms [12–16], as well as for gene and cell therapy [17–19]. We have also employed CRISPR to engineer the PCC 7942 genome [20] and effectively inserted DNA fragments as large as 7 kb into *Escherichia coli* genome [21].

Furthermore, the catalytic domains of Cas9 are mutated to generate the inactive Cas9 (dCas9) lacking the endonuclease activity. dCas9 is used in conjunction with the chimeric single guide RNA (sgRNA) wherein the mature crRNA is fused to a partial tracrRNA to mimic the natural crRNA:tracrRNA duplex. By co-expression, the dCas9-sgRNA complex specifically binds to the target gene at the promoter or coding sequence and acts as a roadblock to the elongating RNA polymerase, hence aborting transcription initiation or elongation [22]. This new technology, termed CRISPR interference (CRISPRi), was recently repurposed to repress sequence-specific genes in diverse eucaryotic and procaryotic cells, for rewiring metabolic networks [23, 24] and high-throughput interrogation of genome-wide gene functions [25, 26]. Very recently, CRISPRi has also exploited for gene regulation in cyanobacteria *Synechcocystis* sp. PCC 6803 [27] and *Synechococcus* sp. PCC 7002 [7]. However, whether CRISPRi functions in PCC 7942 has yet to be explored.

In this study, we harnessed the CRISPRi system to effectively knockdown exogenous and endogenous genes in PCC 7942 via appropriate sgRNA design. Selective repression of *glgc*, *sdhA* and *sdhB* genes increased the succinate production by PCC 7942, hence demonstrating the feasibility of employing CRISPRi for the metabolic engineering of PCC 7942 and production of bio-derived chemicals.

## Methods

### Microorganisms

All molecular cloning experiments were performed using *E. coli* DH5α strain (Sigma). Unless otherwise noted, for suspension culture *S. elongatus* PCC 7942 (Invitrogen) was cultivated in a 250 ml shake flask containing 40 ml BG-11 medium [4, 5] with or without antibiotics (gyratory shaking at 100 rpm, with sterile air containing 0.04% CO_2_) in a 30 °C incubator (600SR, Hipoint) with illumination from continuous cool white fluorescent light (intensity ≈ 70 μmol//m^2^ s). For solid culture, PCC 7942 cells were streaked onto 90 mm plates containing 40 cm^3^ BG-11/agar medium supplemented with 1 mM sodium thiosulfate and appropriate antibiotic, and incubated with continuous illumination (intensity 70 µmol//m^2^ s) for 7–9 days until colonies developed.

### Plasmids construction

pdCas9-bacteria plasmid (Addgene #44249) harbored chloramphenicol resistance gene (Cm^R^) and *dCas9* gene (derived from *S. pyogenes*) driven by the P_LtetO1_ promoter [24]. The sequences homologous to the 5′ (5-NSI) and 3′ (3-NSI) end of PCC 7942 NSI site (neutral site I), together with the intervening origin of replication (ori), were PCR-amplified from pSYN_1 plasmid (Invitrogen) with flanking *Avr*II and *Spe*I sites. pdCas9-bacteria and the PCR product were separately digested with *Avr*II/*Spe*I and ligated together (Additional file 1: Figure S1). The resultant pLtetO1-dCas9 contained the Cm^R^ and P_LtetO1_-dCas9 expression cassettes flanked by the 5-NSI and 3-NSI homology arms (Additional file 1: Figure S1).

We next PCR-amplified the P_smt_ promoter (including the promoter smtA and the repressor smtB) from the PCC 7942 chromosome, which was cloned into pLtetO1-dCas9 by *Afl*II/*Bgl*II digestion to replace the P_LtetO1_ promoter, yielding pSdCas9 (Additional file 1: Figure S2). The *eyfp* gene under the control of P_conII_ promoter was PCR-amplified from pconII-EYFP’ (see Additional file 1: Supplementary Methods) and subcloned into pSdCas9 by *Avr*II/*Sma*I (Additional file 1: Figure S3). The resultant pSdCas9-CY’ harbored the expression cassettes comprising Cm^R^, *dCas9* under P_smt_ and *eyfp* under P_conII_, which were flanked by homology arms targeting the NSI site (5-NSI and 3-NSI).

pgRNA-bacteria plasmid (Addgene, #44251) contained the ampicillin-resistance gene (Ap^R^) and an sgRNA backbone driven by P_J23119_ promoter. The sgRNA backbone comprised the base-pairing (spacer) region (20 bp), dCas9 handle (42 bp) and the *S. pyogenes* terminator (40 bp) as described [22]. To replace the spacer sequence on the sgRNA backbone with new spacer sequences targeting different regions on the PCC 7942 chromosome, we designed a reverse primer Ec_R and forward primers Ec_F with different new spacer sequences (Table 1), and performed inverse PCR (iPCR) using pgRNA-bacteria as the template [22]. The resultant PCR products comprising the new sgRNA sequences, Ap^R^ and P_J23119_ promoter were phosphorylated using T4 polynucleotide kinase and joined using T4 DNA ligase to form new plasmids (bacteria sgRNA plasmids) containing sgRNA targeting different regions on the PCC 7942 chromosome (Additional file 1: Figure S4A).

**Table 1:** Primer sequences for psgRNA plasmid construction

Primers	Sequence (5′–3′)
Primers for sgRNA spacer to target *eyfp*	
Ec-Φ_F	GTTTTAGAGCTAGAAATAGCAAGTTAAAATAAGGC
Ec-P1_F	ATTAATTGTCAATTCGAAACGTTTTAGAGCTAGAAATAGCAAGTTAAAATAAGGC
Ec-NT1_F	CCGTCCAGCTCGACCAGGATGTTTTAGAGCTAGAAATAGCAAGTTAAAATAAGGC
Ec-NT2_F	GCGCTCCTGGACGTAGCCTTGTTTTAGAGCTAGAAATAGCAAGTTAAAATAAGGC
Ec_R	ACTAGTATTATACCTAGGACTGAGCTAGC
Primers for sgRNA spacer to target *glgc, sdhA* and *sdhB*	
Ec-glgc1_F	TTGGCGCGCTGTTTGGTTAGGTTTTAGAGCTAGAAATAGCAAGTTAAAATAAGGC
Ec-glgc2_F	AGAGGTTGTAGGTCTGACTGGTTTTAGAGCTAGAAATAGCAAGTTAAAATAAGGC
Ec-sdhA1_F	TAATCAACGGCAATGTGTCAGTTTTAGAGCTAGAAATAGCAAGTTAAAATAAGGC
Ec-sdhA2_F	GCCCTGAGCCGCCACGCTATGTTTTAGAGCTAGAAATAGCAAGTTAAAATAAGGC
Ec-sdhB1_F	AGATCGTGACTGCAGGAATAGTTTTAGAGCTAGAAATAGCAAGTTAAAATAAGGC
Ec-sdhB2_F	GCCGCCAAATCTTAAATTTCGTTTTAGAGCTAGAAATAGCAAGTTAAAATAAGGC
Ec_R	ACTAGTATTATACCTAGGACTGAGCTAGC

The spacer sequences are underlined

To integrate the sgRNA sequences into NSII (neutral site II) site, the sequences homologous to the 5′ (5-NSII) and 3′ (3-NSII) end of PCC 7942 NSII site were PCR-amplified from pNSII_plus plasmid (kindly provided by Prof. James Liao). The resultant PCR product consisted of 5-NSII, origin of replication (ColE1), 3-NSII and kanamycin resistance gene (Km^R^). The PCR product and bacteria sgRNA plasmid were digested by *Eco*RI/*Bam*HI and ligated to form the psgRNA plasmids as shown in Additional file 1: Figure S4B. The resultant plasmids were designated as psgRNA::Φ, psgRNA::P1, psgRNA::NT1, psgRNA::NT2, psgRNA::glgc1, psgRNA::glgc2, psgRNA::sdhA1, psgRNA::sdhA2, psgRNA::sdhB1 or psgRNA::sdhB2, depending on the target gene and location (see “Results” section).

### Transformation and recombinant cell construction

For transformation into PCC 7942, 40 ml cells in the shake flask were cultured to optical density at 730 nm (OD_730_) = 0.6–0.8, centrifuged (5000×*g* for 15 min), washed with 20 ml BG-11 medium, centrifuged again, resuspended in 2 ml BG-11 medium and aliquoted (300 µl per microfuge tube). The plasmids were quantified using Nanodrop 2000 (Thermo), and 2000 ng plasmid was added to the tubes and mixed well with the aliquoted cells. The tubes were wrapped with foil and incubated with the rotary mixer in the incubator (30 °C) for 24 h to enhance the transformation efficiency as described [28]. The transformed cells were streaked onto the BG-11/agar plate containing appropriate antibiotics (e.g. 5 µg/ml Cm and 10 µg/ml Km) and cultured until colonies developed. The colonies were re-streaked twice onto the BG-11/agar plate containing antibiotics to yield the recombinant cells.

### Growth curve, flow cytometry and confocal microscopy

The recombinant cells were transferred to and cultured in the shake flask containing Cm/Km. In parallel, wild-type (WT) cells were cultured in the same fashion without antibiotics. One milliliter of cells were sampled daily for the measurement of OD_730_ for the growth curve. The cells (1 ml) were also withdrawn every 3 days for EYFP analysis, followed by replenishment with 4 ml fresh BG-11 medium with or without antibiotics.

For *eyfp* expression analysis, the cells were subjected to flow cytometry (FACSCalibur, BD Biosciences) and the mean fluorescence intensity (FI) of 10,000 cells was measured. The mean FI (in arbitrary unit, a.u.) of each group was subtracted from that of WT cells to yield the final mean FI.

Alternatively, the *eyfp*-expressing recombinant cells were cultured in the shake flask to mid-log phase (OD_730_ = 1–1.5), and the cells were subjected to flow cytometry analysis or observed at 1000× under a confocal microscope (Eclipse TE2000-E, Nikon) for the yellow fluorescence (488 nm) and auto fluorescence (543 nm).

### Quantification of mRNA by qRT-PCR

PCC 7942 cells were cultured in 40 ml BG-11 medium with or without antibiotics to OD_730_ = 0.7–1.3 and 5 ml cells were centrifuged (17,000×*g* for 5 min) and stored at −80 °C. After thawing, total RNA was extracted using NucleoSpin® RNAIIKit (Macherey Acherey-Nagel) with minor modifications to increase the extraction efficiency: the lysozyme concentration was increased to 2 mg/ml and reaction time at 37 °C was extended to 20 min. The extracted mRNA was quantified using a spectrophotometer (Nanodrop 2000, Thermo) and 1 µg RNA was reverse-transcribed to cDNA using the MMLV Reverse Transcription 1st-strand cDNA Synthesis Kit (Epicentre Biotechnologies). The cDNA was diluted in 1 ml deionized water and stored at −20 °C. After thawing, 3 µl cDNA was mixed with 1.5 µl deionized water, 0.5 µl gene-specific primer pairs (10 µM, Table S1) and 5 µl SYBR® Green PCR Master Mix (Applied Biosystems). Subsequent quantitative real-time PCR (qPCR) was performed using StepOnePlus™ (Applied Biosystems) with the PCC 7942 housekeeping gene *rnpB* as the internal control [29]. Gene expression levels in all groups were normalized to those in the WT control group.

### Glycogen analysis

Because PCC 7942 accumulates glycogen under nitrogen starvation conditions [30], we cultured cells in nitrate-deplete (0×N) BG-11 medium (which is similar to BG-11 medium except that NaNO_3_ was not included) to force cells to accumulate glycogen. Recombinant PCC 7942 cells cultured in shake flasks to mid-log phase (OD_730_ ≈ 0.7–1.3) were centrifuged (25 °C, 6000×*g* for 20 min), resuspended in 20 ml nitrate-deplete (0×N) BG-11 medium, centrifuged again, resuspended in 40 ml nitrate-deplete BG-11 medium containing Km/Cm, and cultured for 2 more days. As a control, WT cells were cultured and washed in a similar fashion, and cultured in nitrate-deplete, antibiotic-free BG-11 medium for 2 more days.

The cell density was adjusted to OD_730_ = 1 and 1 ml of cells were centrifuged (17,000×*g* for 5 min), followed by resuspension in 1 ml deionized water and homogenization using Bead Beater (Kelowna). After centrifugation (17,000×*g*, 5 min), the glycogen content (µg) in the supernatant was analyzed using the Glycogen Colorimetric/Fluorometric Assay Kit (Biovision, k646-100).

### Succinate analysis

Recombinant PCC 7942 cells cultured in the shake flasks to stationary phase (OD_730_ ≈ 2.0) were centrifuged (25 °C, 6000×*g* for 20 min), and resuspended in 20 ml nitrate-deplete (0×N) BG-11 medium. After centrifugation, the cells were resuspended in 40 ml nitrate-deplete BG-11 medium containing Km/Cm and cultured in the shake flasks.

As a control, WT PCC 7942 cells were cultured in 80 ml BG-11 medium to OD_730_ ≈ 2.0, divided in half into 50 ml tubes, washed twice by centrifugation (6000×*g*, 20 min) and resuspension in 40 ml complete or nitrate-deplete BG-11 medium, followed by shake flask culture using 40 ml complete (1×N) or nitrate-deplete (0×N) BG-11 medium.

After 2 days, 1.5 ml recombinant or WT cells were collected, filtered through 0.22 µm and the supernatant was analyzed by UFLC-MS (LCMS-2020, Shimadzu) with a column [Acclaim™ Organic Acid, 3 µm, 2.1 × 150 mm (Dionex)] for the succinic acid titer (mg/l).

### Statistical analysis

All quantitative data were analyzed using student’s *t* test. All data represent the averages of at least 3 independent culture experiments. *p* < 0.05 was considered significant.

## Results

### Pre-test of promoters for gene expression in *S. elongatus* PCC 7942

Although a number of inducible promoters (e.g. high-light-responsive p_sbA_ promoter, copper-regulated p_etE_ promoter, nitrate/nitrite-inducible P_nirA_ promoter and the nickel-regulated nrsA promoter) have been assessed in cyanobacteria, induction of these regulatory systems could concurrently affect the endogenous cognate regulon and result in unwanted effects [1, 31, 32]. To evaluate appropriate promoters useful for driving CRISPRi in PCC 7942, we compared different inducible promoters (P_smt_, P_LtetO1_, P_ConII-ribo_, P_trc_, P_LlacO1_ and P_BAD_) derived from *E. coli* or cyanobacteria (Additional file 1: Figure S5). We determined that P_smt_ gave the highest enhanced yellow fluorescent protein (EYFP) expression and the highest induction ratio in PCC 7942 (Additional file 1: Figure S6). We next assessed various constitutive promoters (P_trc’_, P_LlacO1′_, P_conII_, P_J23101_ and P_J23119_, Additional file 1: Figure S7) and determined that P_conII_ and P_J23119_ gave rise to the highest *eyfp* expression levels (Additional file 1: Figure S8).

### Establishment of CRISPRi system in PCC 7942

**Fig. 1:**
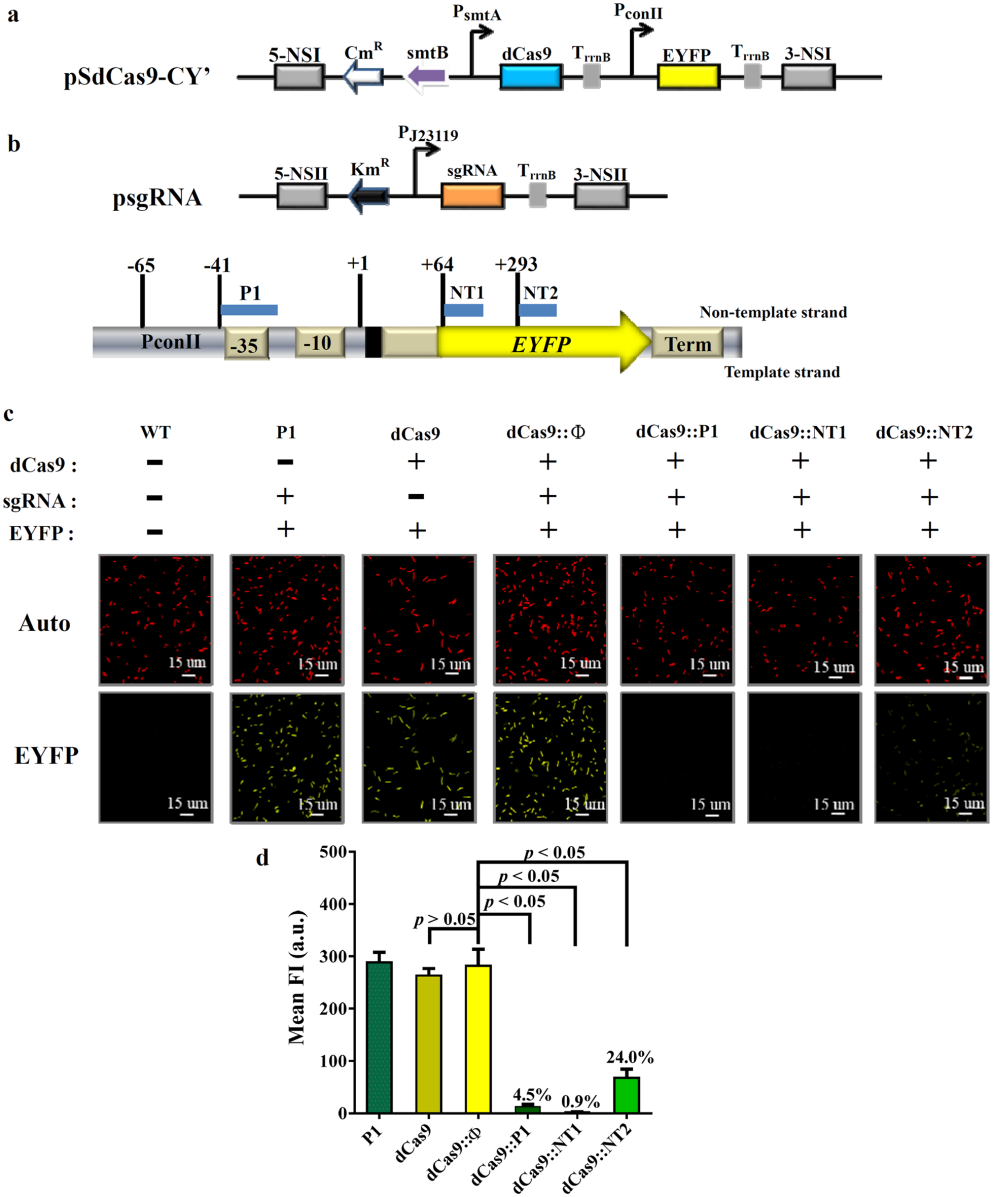
Establishment of CRISPRi in PCC 7942. **a** Map of pSdCas9-CY’ harboring Cm^R^, *dCas9* under P_smt_ and *eyfp* under P_conII_, which was flanked by homology arms targeting the NSI site. T_rrnb_, transcriptional terminator. P_smt_ consists of the smtA promoter and the smtB repressor. **b** Map of psgRNA harboring the cassette expressing Km^R^ and sgRNA under the P_J23119_ promoter, which was flanked by NSII-targeting homology arms. The sgRNAs were designed to target no sequence on the PCC 7942 chromosome (Φ) or the *eyfp* cassette at the non-template strand of promoter (P1) or coding regions near the transcription start site (NT1 and NT2). The *numbers* indicate the position relative to the transcription start site. **c** Confocal micrographs of cells. **d** Flow cytometry analysis data. pSdCas9-CY’ was first transformed into PCC 7942 for cassette integration into NSI site, re-streaked, then individual psgRNA was transformed into the recombinant cells for integration into NSII site. The transformants colonies were transferred to shake flasks and cultured to OD_730_ = 1–1.5 in BG-11 medium containing appropriate antibiotics

In light of aforementioned data, these 3 promoters were chosen for subsequent establishment of CRISPRi system in PCC 7942. We constructed pSdCas9-CY’ with the expression cassette comprised of chloramphenicol resistance gene (Cm^R^), *dCas9* under P_smt_, and *eyfp* under P_conII_, which was flanked by homology arms targeting the NSI site (Fig. 1a). In parallel, we constructed a series of psgRNA plasmids harboring the cassette expressing kanamycin resistance gene (Km^R^) and sgRNA under the P_J23119_ promoter, which was flanked by NSII-targeting homology arms (upper panel, Fig. 1b). The sgRNA were designed to target the *eyfp* cassette at the non-template strand of promoter (P1) or coding regions near the transcription start site (NT1) or near the middle of gene (NT2) so that the fluorescence intensity served as the indicator of gene repression (lower panel, Fig. 1b). We transformed pSdCas9-CY’ into PCC 7942 for cassette integration into NSI site, re-streaked, and then transformed individual psgRNA into the recombinant cells for integration into NSII site (Additional file 1: Figure S9), yielding recombinant clones expressing EYFP, dCas9 and different sgRNA (dCas9::P1, dCas9::NT1 or dCas9::NT2). As controls, we also constructed cells that expressed (1) only EYFP and the sgRNA targeting P1 (P1 group); (2) dCas9 and EYFP (dCas9 group) and (3) dCas9, EYFP and a scramble sgRNA (dCas9::Φ group). The cells were streaked onto BG-11/agar plates containing Km/Cm for 7–9 days, and resistant (Km^R^/Cm^R^) colonies were picked for colony PCR to verify correct integration (data not shown). The colonies were transferred to shake flasks and cultured to OD_730_ = 1–1.5 in BG-11 medium containing appropriate antibiotics.

The fluorescence micrographs (Fig. 1c) showed that all groups had similar auto-fluorescence and the 3 control groups (P1, dCas9 and dCas9::Φ) lacking complete functional dCas9/sgRNA complex exhibited similar EYFP expression. In contrast, EYFP expression was diminished in the 3 experimental groups (dCas9::P1, dCas9::NT1 and dCas9::NT2) expressing both dCas9 and sgRNA (Fig. 1c). The flow cytometry analysis (Fig. 1d) further depicted that EYFP expression in the dCas9::P1, dCas9::NT1 and dCas9::NT2 was suppressed to ≈4.5, ≈0.9 and ≈24.0% that of the control dCas9::Φ group, respectively. These data confirmed the successful establishment of CRISPRi system that effectively repressed gene expression for up to ≈111-fold in PCC 7942. It is noteworthy that dCas9 was driven by an inducible promoter P_smt_ which could be induced with ZnSO_4_. However, even without inducer, the EYFP repression was still very effective (Fig. 1c), suggesting that a low basal level of dCas9 expression was sufficient to mediate the gene suppression. Therefore, the inducer was not added in all subsequent experiments.

### Effect of CRISPRi on PCC 7942 growth and persistent transgene repression

**Fig. 2:**
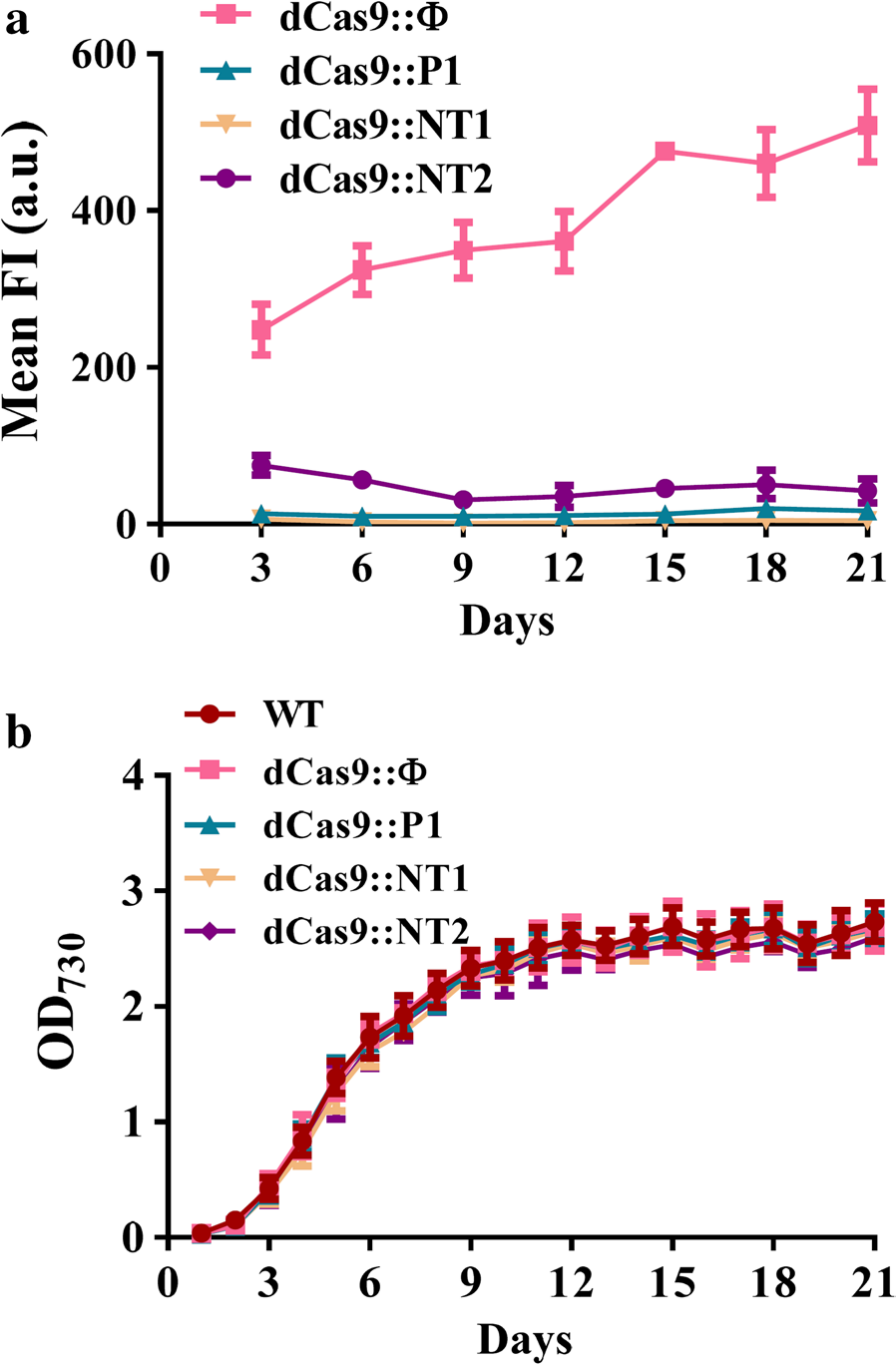
Effect of CRISPRi on PCC 7942 growth and persistent transgene repression. **a** Mean fluorescence intensity (FI) profiles. **b** Growth curves. The recombinant cells were cultured in 40 ml BG-11 medium containing Km/Cm. Wild-type (WT) cells were cultured in 40 ml BG-11 without antibiotics. The cells were cultured for 21 days, during which cells were sampled for OD_730_ or flow cytometry analysis

To examine whether CRISPRi conferred long-term, stable gene suppression and imposed toxicity, the Km^R^/Cm^R^ colonies of all groups were transferred to shake flasks and cultured in 40 ml BG-11 medium containing Km/Cm. As a control, wild-type (WT) cells were cultured in 40 ml BG-11 without antibiotics. The cells were cultured for 21 days, during which cells were sampled for OD_730_ or flow cytometry analysis. As judged from the flow cytometry analysis (Fig. 2a), dCas9::Φ continued to express EYFP throughout the experiment, whereas the EYFP expression in the dCas9::P1, dCas9::NT1 and dCas9::NT2 groups remained suppressed for 21 days, proving that CRISPRi was able to persistently knockdown gene expression in PCC 7942. Meanwhile, all groups, including the WT control, had virtually overlapped growth curves (*p* > 0.05, Fig. 2b), indicating that dCas9 expression and persistent sgRNA expression did not affect the cell growth.

### CRISPRi-mediated suppression of endogenous genes

Cyanobacteria can accumulate glycogen as a carbon sink under nitrogen starvation conditions. Deleting the *glgc* gene can abolish glycogen synthesis [33, 34] because glgc gene product is a key enzyme in the glycogen synthesis pathway. Conversely, *sdhA* and *sdhB* gene products are responsible for converting succinate to fumarate in the TCA cycle.

**Fig. 3:**
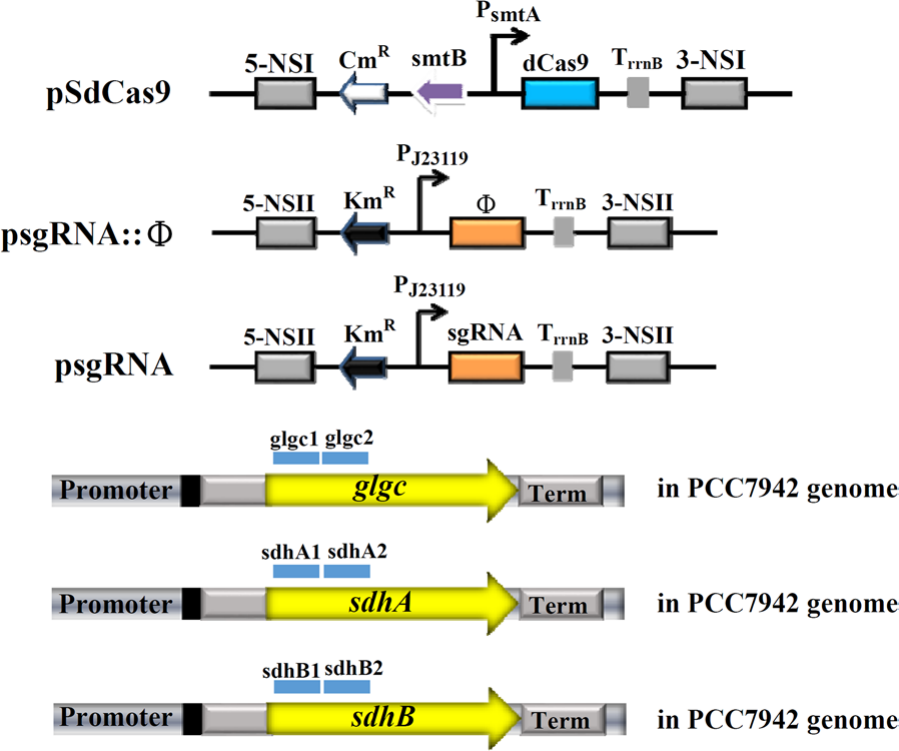
Schematic illustration of plasmids used for CRISPRi-mediated suppression of *glgc*, *sdhA* and *sdhB*. Φ indicates the scramble sgRNA. Glgc1, glgc2, sdhA1, sdhA2, sdhB1 and sdhB2 are the sgRNA targeting different regions of endogenous genes. pSdCas9 expressed dCas9 under P_smt_

To evaluate the ability of CRISPRi to suppress endogenous genes, we constructed pSdCas9 that expressed dCas9 under P_smt_ and a new series of psgRNA that targeted no sequences on the genome of PCC 7942 (psgRNA::Φ) or different coding regions of *glgc*, *sdhA* and *sdhB* genes (Fig. 3a). PCC 7942 cells were transformed with pSdCas9 first for integration into NSI site, and were transformed again with individual psgRNA plasmids for integration into NSII site. The Km^R^/Cm^R^ colonies were transferred to shake flasks and cultured to OD_730_ = 0.7–1.3, and 5 ml cells were sampled for mRNA analysis.

**Fig. 4:**
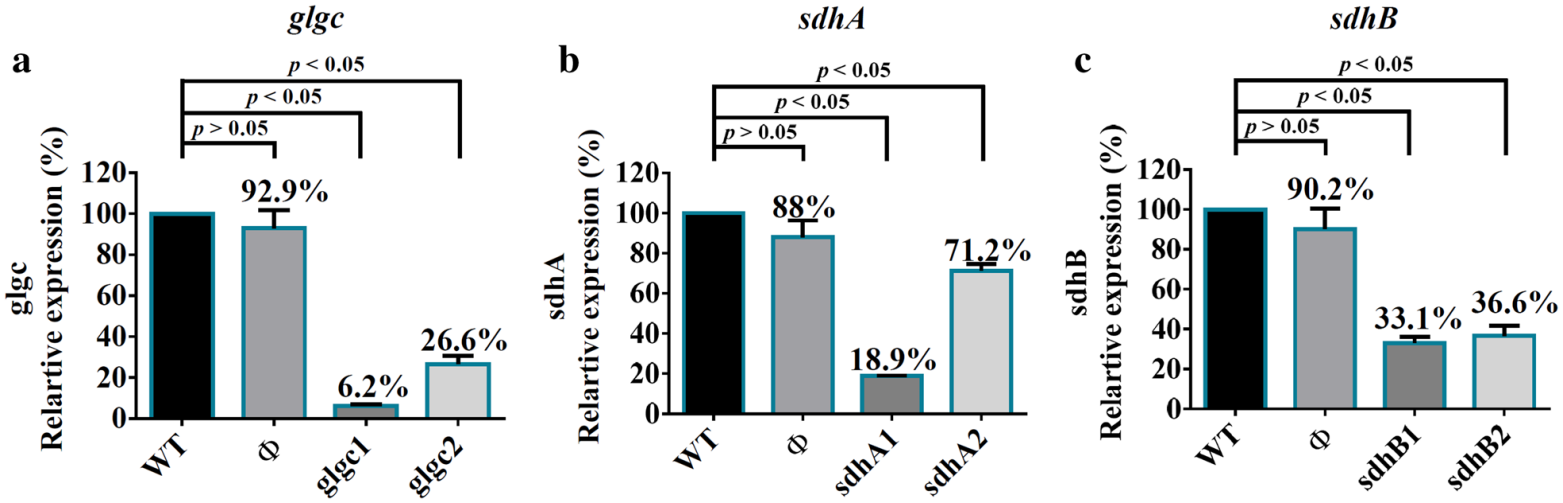
Effective suppression of endogenous genes by CRISPRi. **a** *glgc* expression. **b** *sdhA* expression. **c** *sdhB* expression. PCC 7942 cells were transformed with pSdCas9 first for integration into NSI site, and were transformed again with individual psgRNA plasmids for integration into NSII site. The Km^R^/Cm^R^ colonies were transferred to 40 ml BG-11 medium containing Km/Cm and cultured to OD_730_ = 0.7–1.3, and 5 ml cells were sampled for qRT-PCR analysis. The expression levels were normalized to those in the WT cells

The qRT-PCR analysis (Fig. 4a–c), using the expression in WT cells as the basis, showed that the scramble sgRNA (Φ) did not significantly (*p* > 0.05) affect the expression of *glgc*, *sdhA* or *sdhB*. In contrast, the sgRNA targeting the non-template coding regions of *glgc* (glgc1 and glgc2) attenuated the *glgc* expression to ≈6.2 and ≈26.6%, respectively (Fig. 4a), which represented ≈16.1- and ≈3.8-fold repression, respectively. The sgRNA targeting *sdhA* (sdhA1 and sdhA2) and *sdhB* (sdhB1 and sdhB2) mitigated the expression to ≈18.9, ≈71.2, ≈33.1 and ≈36.6%, respectively (Fig. 4b, c). We also designed sgRNA targeting further downstream of the coding regions and found poorer repression efficiency (data not shown). These data attested that CRISPRi effectively suppressed the endogenous gene expression, and the silencing efficacy was inversely correlated with the distance of target region from the transcription start site.

### CRISPRi-mediated gene downregulation enhanced the production of chemicals

**Fig. 5:**
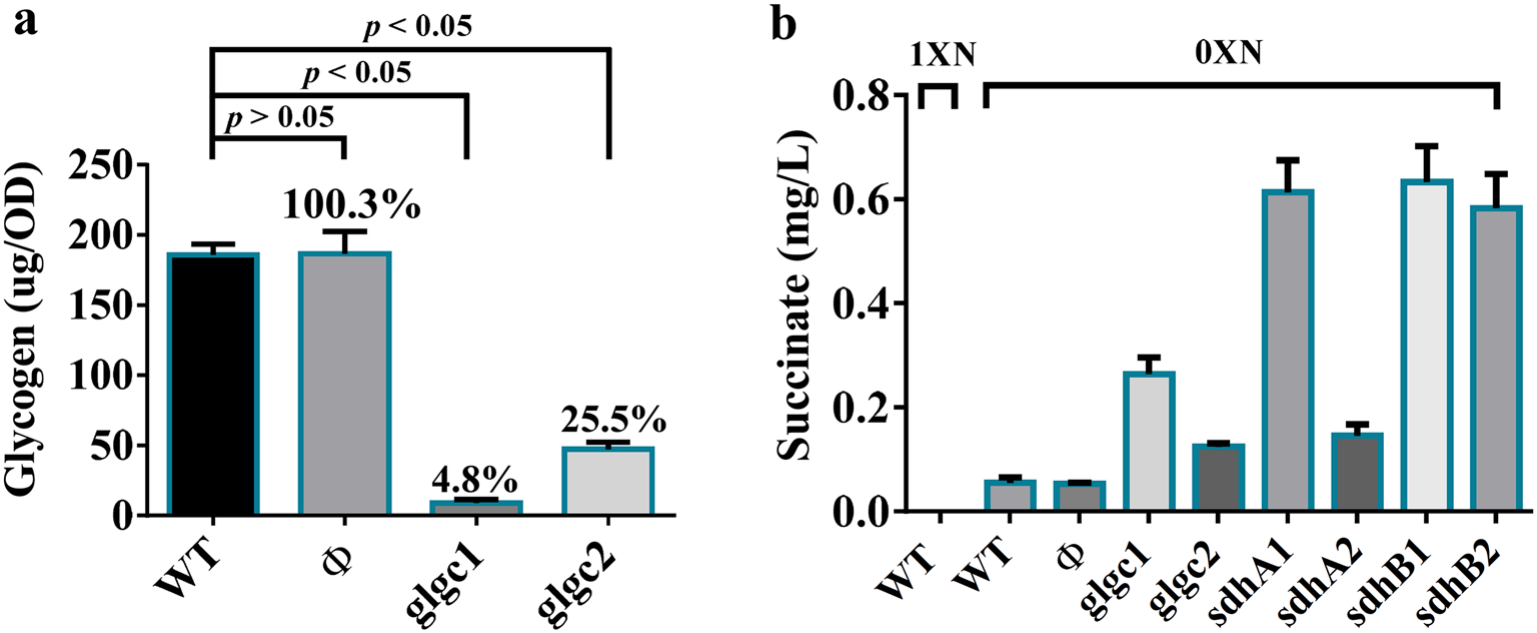
Effects of CRISPRi-mediated suppression on glycogen accumulation and succinate production. **a** Glycogen titer (µg/OD_730_). **b** Succinate titer (mg/l). For glycogen analysis, Km^R^/Cm^R^ cells were cultured to OD_730_ ≈ 0.7–1.3, centrifuged and cultured in shake flasks containing 40 ml nitrogen-deplete (0×N) BG-11 for 2 days and subjected to glycogen analysis. To analyze succinate titer, PCC 7942 cells cultured in shake flasks to stationary phase (OD_73_ ≈ 2.0) were centrifuged, and resuspended in 20 ml nitrate-free BG-11 medium. After centrifugation, the cells were resuspended in 40 ml nitrate-deplete (0×N) BG-11 medium containing Km/Cm and cultured in shake flasks. The WT cells were cultured in the nitrate-deplete (0×N) BG-11 or BG-11 (nitrate-replete) (1×N) medium

To evaluate the effect of repressing *glgc*, *sdhA* or *sdhB* genes, Km^R^/Cm^R^ cells were cultured to OD_730_ ≈ 0.7–1.3, centrifuged and cultured in shake flasks containing 40 ml nitrogen-deplete BG-11 (BG-11 devoid of NaNO_3_) for 2 days. Under nitrogen starvation conditions, suppression of *glgc* gene concomitantly mitigated the glycogen accumulation to ≈4.8% (glgc 1 group) and 25.5% (glgc2 group) that of the WT group (Fig. 5a), but did not induce chlorosis (a condition in which cells produce insufficient chlorophyll, data not shown). We also attempted to target other non-template coding regions of *glgc* further far away from the start codon than glgc1 and glgc2, which attenuated glycogen accumulation with varying degrees (from 37 to 72%, data not shown).

Moreover, we examined the succinate production by individually targeting the aforementioned gene regions and compared the succinate levels with those of the non-targeting control (Φ group) and WT groups under nitrate-replete (1×N) and nitrate-deplete (0×N) conditions (nitrogen-deplete BG-11 medium). Figure 5b delineates that WT cells cultured with nitrogen (1×N) did not produce appreciable amounts of succinate, yet produced slightly more succinate (≈0.05 mg/L) under nitrogen starvation (0×N) conditions. Repression with glgc1, sdhA1, sdhB1 and sdhB2 led to significantly elevated succinate titer when compared with WT cells and the cells without CRISPRi suppression (Φ group), reaching ≈0.26, ≈0.61, ≈0.58 and ≈0.63 mg/L, respectively (Fig. 5b). By suppressing *sdhA* or *sdhB*, the succinate titer (≈0.58–0.63 mg/L) was ≈12.5-fold higher than that in the WT cells. These data collectively confirmed that CRISPRi was able to suppress endogenous genes and enhanced succinate production in PCC 7942.

## Discussion

CRISPRi holds great promise for a wide range of applications in microorganisms, including bacterial cell growth control [35], genetic screen [25, 36], synthetic biology module development [37, 38] or metabolic networks control in various microorganisms such as *E. coli* [24, 39, 40], mycobacteria [41], *Bacillus subtilis* [42], *Corynebacterium glutamicum* [43], *Clostridium beijerinckii* [44], yeast [45] and cyanobacteria [7]. In particular, a number of recent studies have exploited CRISPRi to regulate the metabolic pathways in *E. coli* for enhanced production of various biotechnological products including poly(3-hydroxybutyrate-*co*-4-hydroxybutyrate) [23], terpenoid [8], pinosylvin [46], flavonoid [47] and mevalonate [48]. *Escherichia coli* is a popular host as it has been extensively studied, grows fast, possesses a single chromosome and plenty of genetic engineering toolkits have been developed for *E. coli* engineering. Furthermore, *E. coli* allows the replication and continued presence of plasmids within the cells, which renders easy establishment and maintenance of CRISPRi elements (dCas9 and sgRNA) in *E. coli*.

In contrast, cyanobacteria grow relatively slowly and current knowledge and synthetic biology tool development for cyanobacteria lag far behind those for *E. coli* [49]. Furthermore, many cyanobacteria possess multiple copies of chromosomes and foreign genes need to be integrated and selected, hence making it much more labor-intensive and time-consuming to establish the CRISPRi system in cyanobacteria. To our best knowledge, only 2 very recent studies have employed CRISPRi to engineer cyanobacteria [7, 27]. Yao et al. explored CRISPRi in *Synechcocystis* sp. PCC 6803 to repress formation of carbon storage compounds polyhydroxybutryate and glycogen during nitrogen starvation [27]. Gordon et al. established the CRISPRi system in *Synechococcus* sp. PCC 7002 to repress synthesis of carboxysome essential for CO_2_ concentrating mechanism and downregulate a key node in nitrogen assimilation for enhanced lactate production [7].

In this study, we exploited the CRISPRi system to modulate the gene expression in another common model cyanobacterium PCC 7942, which remarkably differs from PCC 6803 and PCC 7002 in many aspects such as positions in the phylogenetic tree, genome size, chromosome copy number, doubling time and growth conditions [49]. To test the feasibility, we first screened a number of inducible and constitutive promoters that might function in PCC 7942. Based on our preliminary data (Additional file 1: Figures S5–S8), we selected the inducible promoter P_smt_ with lowest leaky expression and highest induction ratio to drive the dCas9 expression, as well as constitutive promoters P_conII_ and P_J23119_ to drive the *eyfp* and sgRNA expression, respectively (Fig. 1a, b). Notably, even without the addition of inducer, the dCas9/sgRNA still suppressed the EYFP expression (Fig. 1c, d). In accord with our observations, Yao et al. employed a panel of P_L_ promoters to drive the dCas9 expression, which however, remarkably repressed the GFP expression in the absence of inducer [27]. Likewise, Gordon et al. employed an anhydrotetracycline (aTc)-inducible promoter to drive dCas9 expression and observed significant EYFP repression in the uninduced state [7]. These findings altogether indicate that leaky expression of dCas9 triggers robust gene perturbation and suggest that a low dCas9 level suffices to mediate CRISPRi-guided suppression.

Such leaky dCas9 expression gave rise to persistent EYFP knockdown (Fig. 2a) without appreciable negative effect on PCC 7942 growth (Fig. 2b), indicating that low level of dCas9 and continued expression of sgRNA do not compromise the health of PCC 7942. This attribute is desirable if the target gene is non-essential for cell growth/metabolism, because such persistent gene repression will obviate the need to add the inducer and save the cost in subsequent large-scale production process. However, if mitigating the target gene expression is detrimental to cell growth and function, an inducible promoter capable of stringent control is necessary. With this regard, Yao et al. have identified a tightly repressed promoter P_L22_ in PCC 6803 that allowed for efficient induction of dCas9 expression with minimal leaky dCas9 expression [27]. Conversely, Gordon et al. engineered the ribosome binding site (RBS) with reduced translation initiation efficiency so as to attenuate the leaky dCas9 expression in PCC 7002. Furthermore, a suite of homologous and heterologous inducible promoters (e.g. P_*idiA*_, P_*isiAB*_, P_etE_, P_nrsB_, etc.) have been developed/explored for their applicability in cyanobacteria [1, 31, 49, 50]. Future studies will be directed towards developing synthetic parts/circuits for tightly regulatable dCas9 expression and robust sgRNA expression in PCC 7942.

Meanwhile, here we designed sgRNA to target different regions of *eyfp* cassette and observed that targeting the regions near -35 of the promoter (P1) and the non-template coding sequence near the transcription start site (TSS) (NT1 and NT2) give rise to effective expression suppression. The data suggested that targeting the promoter and a region as close to the TSS as possible gave better suppression, which agreed well with the sgRNA design rule proposed previously for *E. coli* [22] and cyanobacteria PCC 6803 [27].

Given the successful repression of exogenous reporter gene, we further employed the same sgRNA design rule to selectively knockdown genes essential for glycogen accumulation (*glgc*) and succinate conversion to fumarate (*sdhA* and *sdhB*) (Fig. 3). Our data demonstrated successful downregulation of these genes using a single sgRNA targeting the coding regions, with sgRNA closer to the TSS resulting in more effective suppression (Fig. 4). In particular, repressing *glgc* using the glgc1 sgRNA effectively suppressed the glycogen accumulation (Fig. 5a) and ameliorated the succinate production titer (Fig. 5b). Conversely, glgc2 sgRNA, which targeted a region further downstream of the TSS gave rise to less effective glycogen accumulation and succinate production (Fig. 5). Likewise, repressing *sdhA* with different sgRNAs (sdhA1 and sdhA2) led to quite different degrees of *sdhA* downregulation (Fig. 4b) and sdhA1 conferred more effective *sdhA* suppression and higher succinate production (Figs. 4b, 5b). Conversely, suppressing *sdhB* with two different sgRNAs (sdhB1 and sdhB2) gave similar degrees of *sdhB* suppression and enhanced succinate production (Fig. 5b). These data altogether confirmed that CRISPRi is able to effectively and precisely suppress the target gene expression and re-direct carbon flux to the desired metabolic product, with positive correlation between the degrees of gene repression and succinate titer. Furthermore, the selected target gene and binding site could profoundly influence the outcome, hence underlining the importance of the sgRNA design.

Successful application of CRISPRi in PCC 7942 for gene knockdown is desirable, because PCC 7942 has been genetically engineered to divert native metabolic pathways for product formation [1, 2]. Traditional engineering approach involves the knockout of chromosomal genes, yet the oligoploidy nature of PCC 7942 makes it difficult to knockout the endogenous gene(s) on all chromosomes at once, hence necessitating time-consuming re-streaking and antibiotic selection. Moreover, knockout of certain genes essential for cell metabolism, survival and/or proper function will impair the ability of the cells to produce the desired product. CRISPRi offers the flexibility to fine-tune the endogenous gene expression levels without completely abrogating the gene functions, hence representing a valuable tool kit to intricately regulate the metabolic flux in the cells. Since CRISPRi can be used for genetic screen with appropriate sgRNA library design [25, 45], CRISPRi also may provide a promising tool to interrogate the functions of genes crucial for cell metabolism and product production in PCC 7942.

## Conclusions

In summary, we demonstrated successful CRISPRi-mediated modulation of gene expression in the cyanobacterium *S. elongatus* PCC 7942. By appropriate sgRNA design, we were able to selectively knockdown exogenous reporter gene (*eyfp*) and endogenous genes (*glgc*, *sdhA* and *sdh*B). Targeted suppression of the endogenous genes involved in succinate synthesis increased the succinate production, with the product titer positively correlating with the degrees of gene suppression. These data demonstrated that CRISPRi enabled customizable RNA-guided, targeted gene suppression, which allowed for re-directing the cellular carbon flow. This study thus paves a new avenue to rationally fine-tune the metabolic pathways in PCC 7942 for the production of biotechnological products.

## Additional file

**Additional file 1.** Supporting information.

## Abbreviations

CRISPR: clustered regularly interspaced short palindromic repeat
CRISPRi: CRISPR interference
dCas9: catalytically inactive Cas9
sgRNA: single guide RNA
EYFP: enhanced yellow fluorescent protein
*S. elongatus*: *Synechococcus elongatus*
crRNA: CRISPR RNA
NSI: neutral site I
Km: kanamycin
Cm: chloramphenicol
Ap: ampicillin
