# Supplementary material for: CRISPR interference (CRISPRi) for gene regulation and succinate production in cyanobacterium S. elongatus PCC 7942
Source: Microb Cell Fact. 2016 Nov 15;15:196. doi: 10.1186/s12934-016-0595-3 (PMC5111286; doi:10.1186/s12934-016-0595-3)
Supplement: Supplementary file 1 — Additional file 1. Supporting information. [file 12934_2016_595_MOESM1_ESM.docx]

**Additional Information**

**Supplementary Methods**

**Construction of plasmids with inducible promoters**

The construction of pLtetO1-dCas9 is described in Materials and Methods and shown in Fig. S1. pLtetO1-dCas9 harbored Cm^R^ and *dCas9* driven by P_LtetO1_ promoter, with flanking sequences homologous to the 5’ (5-NSI) and 3’ (3-NSI) regions of NSI site on PCC 7942.

The *eyfp* gene with or without ribosome binding site (RBS) was PCR-amplified from pEYFP-1 (Clontech), subcloned into pLtetO1-dCas9 by *Bgl*II/*Xho*I digestion to replace *dCas9* gene in order to yield pLtetO1-EYFP’ and pLtetO1-NR-EYFP. The heterologous inducible promoters included P_trc_ ([Atsumi*, et al.*, 2009](#_ENREF_1)), P_LlacO1_ ([Atsumi, et al., 2009](#_ENREF_1); [Lee*, et al.*, 2011](#_ENREF_4)), P_LtetO1_, P_conII-riboswitch_ ([Ma*, et al.*, 2014](#_ENREF_5)) (abbreviated as P_conII-ribo_) and P_BAD_. The endogenous inducible promoter was P*smt* which consisted of the smtA promoter and smtB repressor ([Erbe*, et al.*, 1995](#_ENREF_3)). P_trc_, P_LlacO1_ and P_smt_ and their corresponding repressors were PCR-amplified from pSE380 (Invitrogen), pBbA6c-RFP (Addgene #35287) and PCC 7942 chromosome, respectively, and cloned into pLtetO1-EYFP’ to replace P_LtetO1_ by *Afl*II/*Bgl*II digestion. The resultant plasmids were designated as ptrc-EYFP’, pLlacO1-EYFP’ and psmt-EYFP’, respectively. P_conII-ribo_ was chemically synthesized (MDBio Inc.) and subcloned into pLtetO1-NR-EYFP to replace P_LtetO1_, yielding pconII-ribo-EYFP’. P_BAD_ was PCR-amplified from pKD46 and subcloned into pconII-ribo-EYFP’ by *Asu*II/*Bgl*II digestion to replace P_conII-ribo_, yielding pBAD-EYFP’. pBAD-NR-EYFP. The *eyfp* gene with RBS was PCR-amplified from pEYFP-1, subcloned into pBAD-NR-EYFP by *BglII/XhoI* digestion to replace *eyfp* gene without RBS yielding pBAD-EYFP’.

**Construction of plasmids with constitutive promoters**

The heterologous constitutive promoters included P_conII_ ([Ma, et al., 2014](#_ENREF_5)), P_J23101_ ([Camsund and Lindblad, 2014](#_ENREF_2)), P_J23119_, Ptrc’ ([Atsumi, et al., 2009](#_ENREF_1)) and P_LlacO1’_ ([Atsumi, et al., 2009](#_ENREF_1); [Lee, et al., 2011](#_ENREF_4)). P_trc’_ and P_LlacO1’_ were similar to P_trc_ and P_LlacO1_ except that the lac repressor was removed so that the promoter became constitutive.

Promoters P_conII_, P_J23101_ and P_J23119_ were chemically synthesized and cloned into pLtetO1-EYFP’ to replace P_LtetO1_ by *Afl*II/*Bgl*II digestion, yielding pconII-EYFP’, pJ23101-EYFP’ and pJ23119-EYFP’, respectively. P_trc’_ and P_LlacO1’_ were PCR-amplified from pSE380 and pBbA6c-RFP, and separately subcloned into pLtetO1-EYFP’ by *Afl*II/*Bgl*II digestion to replace P_LtetO1_, yielding ptrc’-EYFP’ and pLlacO1’-EYFP’, respectively.


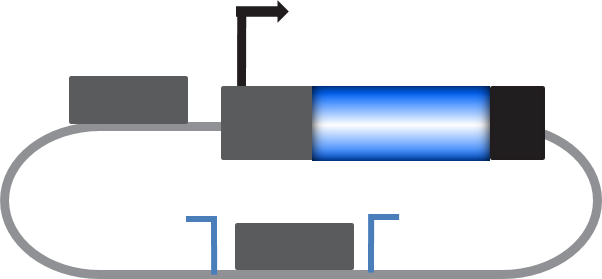

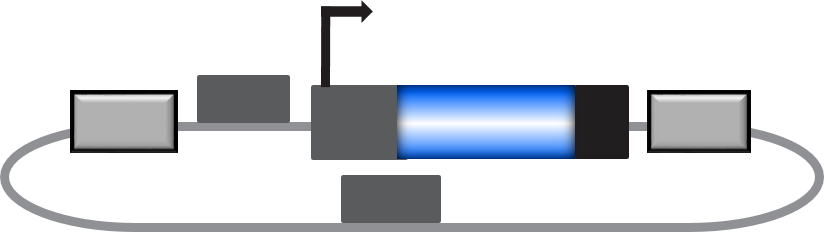


**Supplementary Figures**

**Enzyme digestion by *Spe*I*/Avr*II**

**Enzyme digestion by *Spe*I*/Avr*II**

**Ligation**


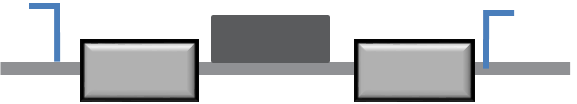


**pdCas9-bacteria**

**P_LtetO1_**

**dCas9**

Cm^R^

p15A

**PCR product from pSYN_1**

**SpeI**

**AvrII**

**SpeI**

**AvrII**

**5-NSI**

**3-NSI**

**5-NSI**

**3-NSI**

pUC

pUC

Cm^R^

**P_LtetO1_**

**pLtetO1-dCas9**

**dCas9**


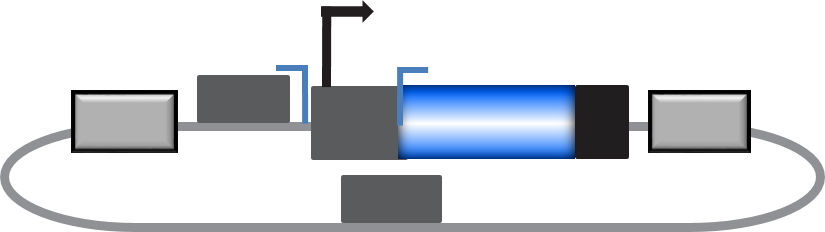

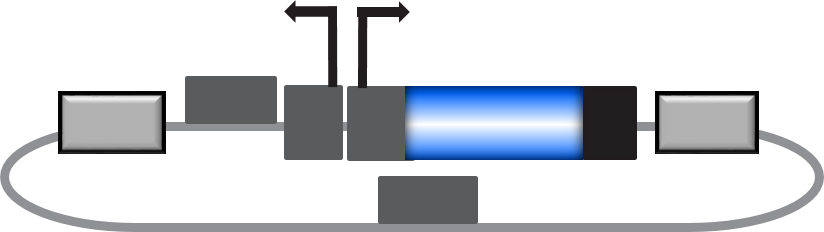


**Fig. S2. Flow chart of pSdCas9 construction.** P_smt_ promoter consists of P_smtA_ and the smtB repressor.

**Enzyme digestion by *Afl*II*/Bgl*II**

**Enzyme digestion by *Afl*II*/Bgl*II**

**Ligation**

**pLtetO1-dCas9**

**P_LtetO1_**

**dCas9**

Cm^R^

pUC

**PCR product from PCC 7942 chromosome**

**AflII**

**BglII**

**BglII**

**AflII**

**5-NSI**

**3-NSI**

pUC

Cm^R^

**P_smtA_**

**pSdCas9**


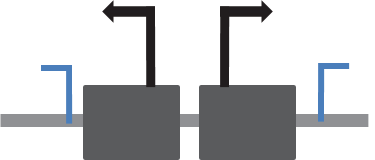


**P_smtB_**

**dCas9**

**P_smtA_**

**P_smtB_**

**3-NSI**

**5-NSI**


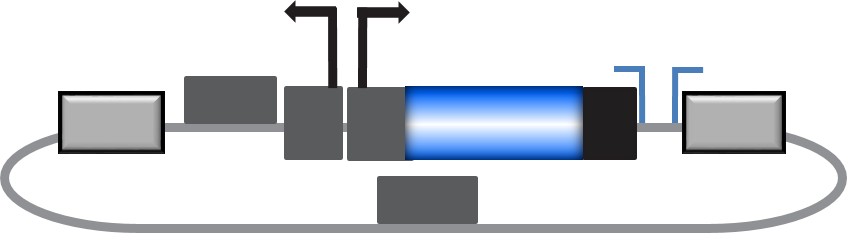

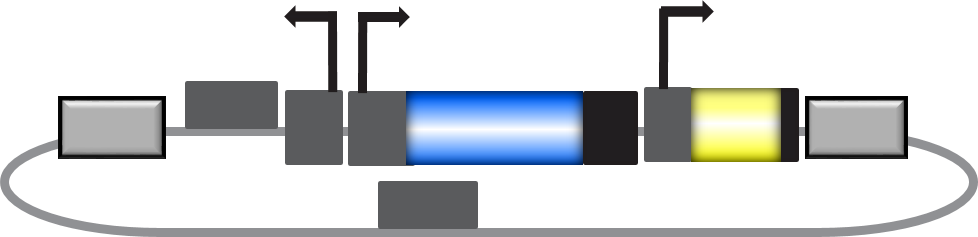


**Fig. S3. Flow chart of pSdCas9-CY’ construction.**

**Enzyme digestion by *Avr*II*/Sma*I**

**Enzyme digestion by *AvrI*I*/Sma*I**

**Ligation**

**pSdCas9**

**P_conII_**

**dCas9**

Cm^R^

pUC

**PCR product from pconII-EYFP’**

**AvrII**

**SmaI**

**SmaI**

**AvrII**

**5-NSI**

**3-NSI**

pUC

Cm^R^

**P_smtA_**

**pSdCas9-CY’**

**P_smtB_**

**dCas9**

**P_smtA_**

**P_smtB_**

**3-NSI**

**5-NSI**


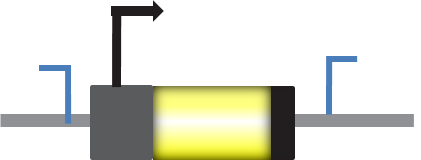


**P_conII_**

**EYFP**

**EYFP**


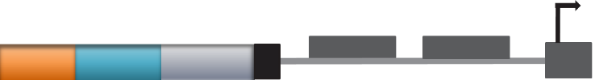


**Fig. S4. Flow chart of psgRNA construction.**

**(A)**

**(B)**

**Enzyme digestion by *EcoRI/BamHI***

**Enzyme digestion by *EcoRI/BamHI***

**Ligation**

**Phosphorylation/ligation**

**PCR product from pNSII_plus**

**psgRNA-bacteria**

**psgRNA**


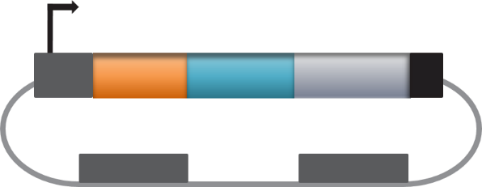


**Bacteria sgRNA plasmid**

**P_J23119_**

**sgRNA**

**20bp**

**42bp**

**40bp**

Ap^R^

ColE1


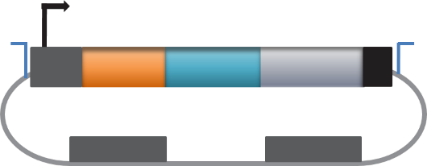


**Bacteria sgRNA plasmid**

**P_J23119_**

**sgRNA**

**20bp**

**42bp**

**40bp**

**EcoRI**

**BamHI**

New spacer

sequence

Ap^R^

ColE1

**P_J23119_**

**sgRNA**

**20bp**

**42bp**

**40bp**

Ap^R^

ColE1


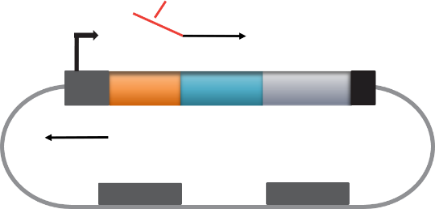


**New spacer region**

Primer Ec-F

**P_J23119_**

**sgRNA**

dCas9

handle

Base-pairing

region

Primer Ec-R

ColE1

Ap^R^

*S. pyogenes*

terminator

Term

(rrnB)

**20bp**

**42bp**

**40bp**

**EcoRI**

**BamHI**

**3-NSII**

**5-NSII**


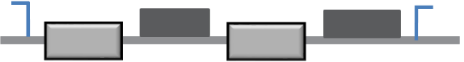


ColE1

Km^R^


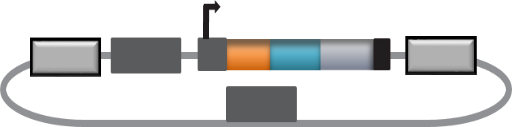


**3-NSII**

**5-NSII**

ColE1

Km^R^

**sgRNA**

**P_J23119_**

**20bp**

**42bp**

**40bp**

**iPCR**

sequence

New spacer

New spacer

sequence

**Fig. S5. Plasmids with EYFP driven by different inducible promoters.** All plasmids were constructed as described in Supplementary Methods and accommodated Cm^R^ and *eyfp* driven by different promoters. The two expression cassettes were flanked by homology arms (5-NSI and 3-NSI). The plasmids (2000 ng) were transformed into PCC 7942 for integration into NSI site of PCC 7942 chromosome. Homologous P_smt_ was derived from PCC 7942, which was repressed by smtB and induced by 8 μM ZnCl_2_. Heterologous promoters derived from *E. coli* included P_trc_, P_LlacO1_, P_LtetO1_, P_conII-riboswitch_ (abbreviated as P_conII-ribo_) and P_BAD_. P_LtetO1_ was repressed by tet^R^ and was induced by 1 μM aTc (anhydrotetracycline). P_conII-ribo_ was repressed by the secondary structure of riboswitch and was induced by 2 mM theophylline. P_trc_ and P_LlacO1_ were repressed by lacI and induced by 1 mM IPTG. P_BAD_ was repressed by araC and was induced by 1 mM arabinose. The inducers were supplemented into medium after we cultured the cells to OD730≈0.6-0.8. The cells were harvested 1 day later for analysis.


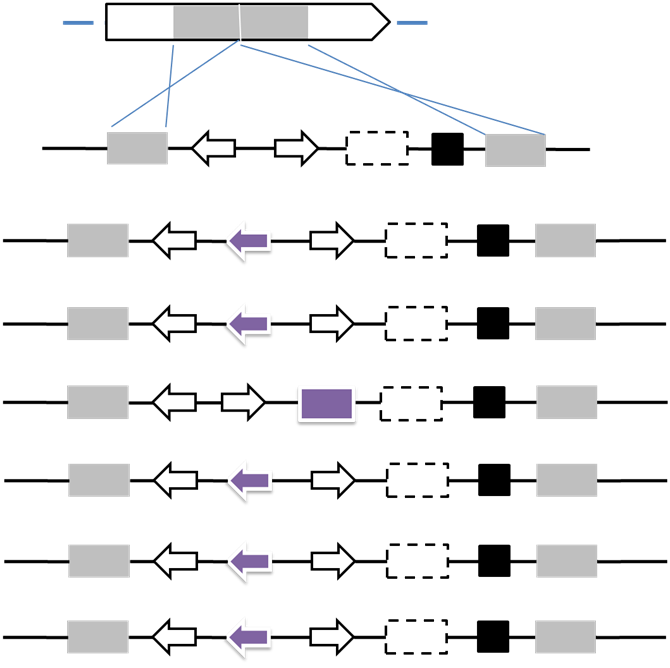


NSI site

PCC 7942 genome

5-NSI

3-NSI

TrrnB

3-NSI

3-NSI

3-NSI

5-NSI

5-NSI

5-NSI

5-NSI

5-NSI

5-NSI

3-NSI

3-NSI

3-NSI

TrrnB

TrrnB

TrrnB

TrrnB

TrrnB

TrrnB

Cm^R^

Cm^R^

Cm^R^

Cm^R^

Cm^R^

Cm^R^

Cm^R^

Promoter

EYFP

P_smtA_

P_trc_

P_BAD_

P_LtetO1_

P_LlacO1_

P_conII_

riboswitch

smtB

tetR

lacI

lacI

araC

EYFP

EYFP

EYFP

EYFP

EYFP

EYFP

- **Inducer**

**1 mM IPTG**

**1 mM IPTG**

**8 µM Zn^2+^**

**2 mM theophylline**

**1 µM aTc**

**1 mM arabinose**

**P_LlacO1_**

**P_trc_**

**P_smt_**

**P_conII-ribo_**

**P_LtetO1_**

**P_BAD_**

**Induction Ratio :**

**6.6**

**2.3**

**2.4**

**2.0**

**5.2**

**1.4**

**Fig. S6. Mean fluorescence intensity of EYFP driven by different promoters.** The plasmids were transformed into PCC 7942 and plated on the BG-11/agar plate containing Cm. After 7-9 days, the colonies were re-streaked twice and transferred to the shake flasks each containing 20 ml BG-11 and chloramphenicol. When the OD_730_ reached 1.0-1.5, the cells were sampled for flow cytometry analysis. The data showed that Zn^2+^-induced P_smt_ promoter resulted in the highest fluorescence intensity (80.3 a.u.), low leaky expression (12.0 a.u.), and hence the highest induction ratio (6.6). P_conII-ribo_ led to the second highest expression level (77.9 a.u.) but the basal level was higher (33.1 a.u.), leading to lower induction ratio (2.4). Other promoters conferred relatively low expression levels.**Fig. S1. Flow chart of pLtetO1-dCas9 construction.**

**uninduced**

**induced**

**Fig. S7. Plasmids with EYFP driven by different constitutive promoters.** All plasmids were constructed as described in Supplementary Methods and accommodated Cm^R^ and *eyfp* driven by different promoters. The two expression cassettes were flanked by homology arms (5-NSI and 3-NSI). The plasmids (2000 ng) were transformed into PCC 7942 for integration into NSI site of PCC 7942 chromosome. P_trc’_ and P_LlacO1’_ were similar to P_trc_ and P_LlacO1_, except that the repressor was not included. P_conII_ was similar to P_conII-ribo_ except that the riboswitch was removed. P_J23101_ and P_J23119_ were constitutive promoters derived from *E. coli*.

PCC 7942 genome

NSI site

Cm^R^

3-NSI

TrrnB

EYFP

TrrnB

3-NSI

5-NSI

3-NSI

3-NSI


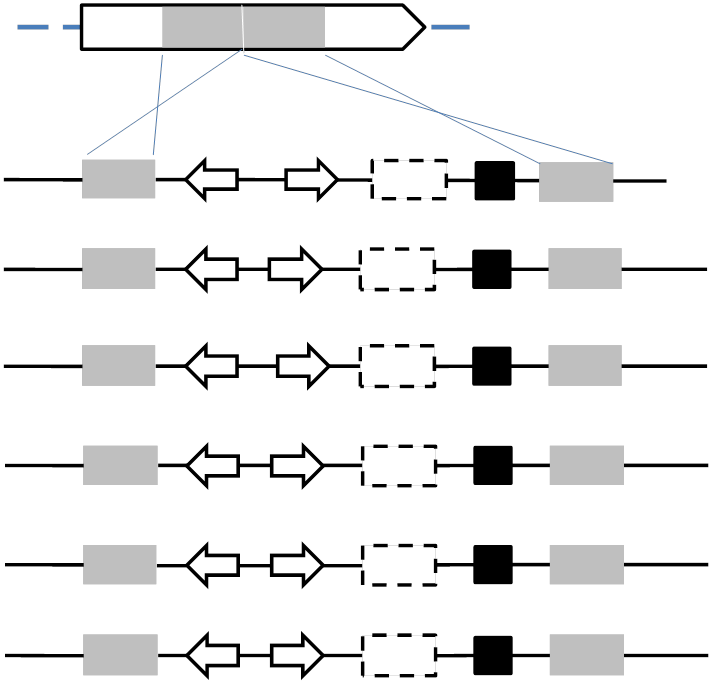


3-NSI

3-NSI

EYFP

EYFP

EYFP

EYFP

EYFP

TrrnB

TrrnB

TrrnB

TrrnB

Cm^R^

Cm^R^

Cm^R^

Cm^R^

Cm^R^

5-NSI

5-NSI

5-NSI

5-NSI

5-NSI

P_trc’_

P_LlacO1’_

Promoter

P_J23119_

P_conII_

P_J23101_

**P_J23101_**

**P_conII_**

**P_trc’_**

**P_LlacO1’_**

**P_J23119_**

**Fig. S8. Fluorescence intensity of EYFP driven by different promoters.** The plasmids were transformed into PCC 7942 and plated on the BG-11/agar plate containing Cm. After 7-9 days, the colonies were re-streaked twice and transferred to the shake flasks each containing 20 ml BG-11 and chloramphenicol. When the OD_730_ reached 1-1.5, the cells were sampled for flow cytometry analysis. The data showed that P_conII_ and P_J23119_ gave rise to the highest EYFP expression, indicating that they are the strongest in PCC 7942.


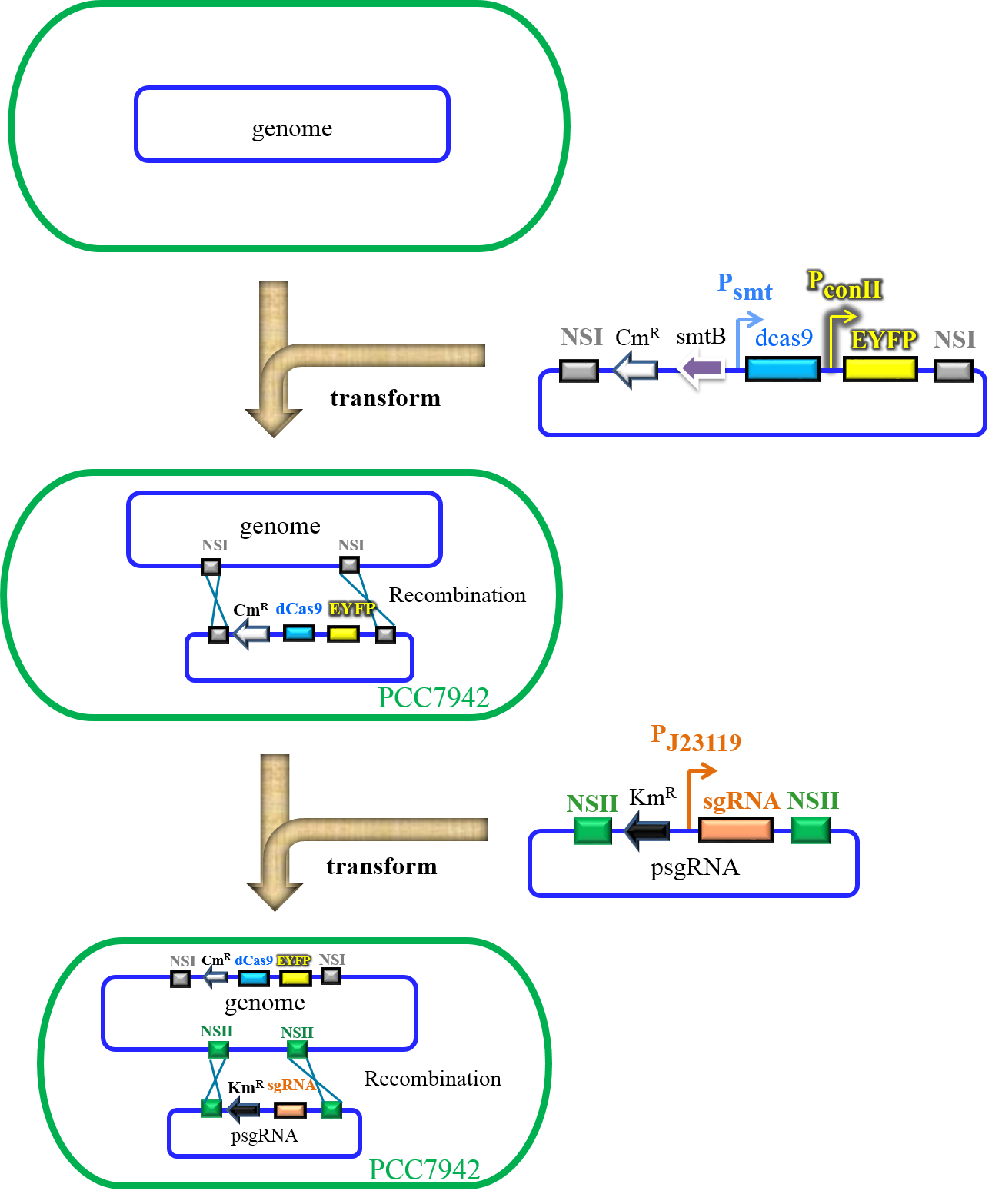


**(B)**

**(A)**

pSdCas9-CY’

pSdCas9-CY’

**Fig. S9. Establishment of CRISPRi system in *S. elongatus* PCC7942*.*** We transformed pSdCas9-CY’ into PCC 7942 for cassette integration into the NSI site, re-streaked, and then transformed individual psgRNA into the recombinant cells for integration into NSII site, yielding recombinant clones expressing EYFP, dCas9 and different sgRNA. pSdCas9-CY’ contained Cm^R^ while psgRNA contained Km^R^, thus the recombinant cells were selected using 5 µg/ml Cm and 10 µg/ml Km.

**Table S1. Gene-specific primer sequence for qRT-PCR**

| Primer | Sequence (5’-3’) |
| --- | --- |
| rnpB-Q F | TGCTTGCAGGCACAGGTAAG |
| rnpB-Q R | CCTCTAGCGGTCCATAAACGG |
| glgc-Q F | GCAAAGTGCCGATGGGAATC |
| glgc-Q R | GGGAATAACCGCCCCTTTGA |
| sdhA-Q F | GGCGAGATTTTGCTAGATGC |
| sdhA-Q R | GGAGCGCTTTCTCTAGCTCA |
| sdhB-Q F | ATCCTCCTGCTTGGATGATG |
| sdhB-Q R | AGCCGGTACCAGTGTTTGTC |

(D)

(C)

**Supplementary References**

Atsumi S, Higashide W, Liao JC. 2009. Direct photosynthetic recycling of carbon dioxide to isobutyraldehyde. Nat Biotechnol 27: 1177-1180.

Camsund D, Lindblad P. 2014. Engineered transcriptional systems for cyanobacterial biotechnology. Front Bioeng Biotechnol 2: 40.

Erbe JL, Taylor KB, Hall LM. 1995. Metalloregulation of the cyanobacterial Smt locus - Identification of Smtb binding sites and direct interaction with metals. Nucleic Acids Res 23: 2472-2478.

Lee TS, Krupa RA, Zhang F, Hajimorad M, Holtz WJ, Prasad N, Lee SK, Keasling JD. 2011. BglBrick vectors and datasheets: A synthetic biology platform for gene expression. J Biol Eng 5: 12.

Ma AT, Schmidt CM, Golden JW. 2014. Regulation of gene expression in diverse cyanobacterial species by using theophylline-responsive riboswitches. Appl Environ Microbiol 80: 6704-6713.
